# Specific mechanisms linking network information processing to the generation of qualia

**DOI:** 10.1093/nc/niaf043

**Published:** 2025-11-06

**Authors:** Roger Orpwood

**Affiliations:** Centre for Pain Research, Department for Health, University of Bath, Combe Down, Bath BA2 7AY, United Kingdom

**Keywords:** Qualia, Hard problem, Neural information processing, Cortical networks

## Abstract

There have been many very promising theories published concerning the generation of consciousness. These theories mostly link the emergence of consciousness to neural activity, but very few attempt to show how that neural activity specifically causes experience to occur. This article explores this problem at the level of individual networks by examining the information changes that occur as input patterns are processed. It looks at how networks can identify spatially distributed input patterns, and generate representations of that identity. It argues that if those representations are directly fed back then such networks will be identifying their own depictions of the original identity. It goes on to argue that in this state the identity acquired is not *what* the input is to the network but *how* the input seems to it. There would be content to that inner portrayal that must present itself in some way to the receiver, and this could underlie the emergence of qualia. The article goes on to argue how top-down modulation could select which qualia are established at any moment.

## Introduction

Most of the promising approaches to deriving theories of consciousness conclude with hypothetical links between the activity of neural structures and the emergence of consciousness. A useful recent review of these theories (Seth and Bayne 2022) shows the range of approaches that have been explored. Most of these approaches explore theories that argue for consciousness to depend on a particular set of neural mechanisms. However, something that is rather lacking is any detailed attempt to explore the specific mechanism whereby conscious awareness is caused, not just in general terms but looking at a specific causal process. Integrated information theory (IIT) argues that there is an equivalence between phenomenological properties of experience and informational/causal properties of physical systems (Tononi et al. 2016). Emergence theories suggest that consciousness emerges from complex interactions between neural components at a higher level of organization (e.g. Feinberg and Mallatt 2019). But the specific process linking neural mechanisms and phenomenal experience still seems elusive. So, irrespective of the general neural processes on which consciousness depends, how does the physical activity occurring within neural tissues directly lead to an experiential outcome, the so-called hard problem of consciousness (Chalmers 1996)?

An interesting set of ideas explored in these general theories argues that conscious perception depends on top-down re-entrant signalling (e.g. Lamme 2006, 2010). Some propose that this re-entrant feedback involves prefrontal areas (Edelman 1992, Lamme and Roelfsema 2000, Bullier 2001, Pascual-Leone and Walsh 2001, Raizada and Grossberg 2003). They argue that information is fed forward through sensory areas and onto the prefrontal cortex, where concluding states are fed back to modulate the incoming sensory activity. The re-entrant activity, as part of attentional focussing, is seen as a key component in the generation of sensory consciousness (Rees et al. 2002, Rees 2007, Lamme 2010). In addition to attentional focussing, some also feel that this re-entrant activity could just be local (Boehler et al. 2008, Lamme 2010). Interaction between higher-level activity and that at a lower level is also a key feature of higher-order theories (HOTs). These theories argue that consciousness depends on metarepresentations of lower-order mental states (e.g. Lau and Rosenthal 2011, Brown et al. 2019). Lower-order representations of sensory activity in the posterior cortex become conscious when activated by certain higher-order metarepresentations. Predictive processing also relies on the active interaction between higher and lower levels of representation to provide a framework for systematically mapping neural mechanisms onto properties of consciousness (Hohwy and Seth 2020).

These general theories involve some kind of higher-level interaction with the processing of information occurring in lower networks. For example, HOT ideas argue for consciousness to arise in a lower-level cortex when targeted by higher-order metarepresentations. Re-entrant theories argue for top-down re-entrant activity being needed to initiate qualia generation in lower-level networks. These ideas seem to point to sensory qualia arising from activity within networks of the sensory cortex itself. Also supporting a local network origin for qualia is the demonstration in the visual cortex that some visual attributes become conscious before others. Zeki showed that conscious perception of colour briefly occurs before that of motion (Moutoussis and Zeki 1997, Zeki and Bartels 1998), which led him to propose the existence of microconsciousness within specific networks in the visual cortex (Zeki 2001).

Therefore, in exploring specific causal mechanisms behind the generation of qualia it might be useful to examine the role of activity in the basic building blocks of cortical information processing, individual cortical networks. This article builds on the author’s previous work on the generation of qualia (Orpwood 2007, 2013, 2017) by exploring the behaviour of cortical networks while they process information, in an attempt to examine in detail the origin of subjective experience.

The article refers to qualia throughout, but in this work ‘qualia’ are meant to imply more than just sensory experiences. Rather, the article uses the more general definition preferred by the philosopher Flanagan, where ‘qualia’ mean any subjective experience (Flanagan 1992), as Flanagan expressed it, ‘anything with a phenomenological feel’. A basic general mechanism for qualia generation could underpin sensory experience, but it could also underpin any other experiential outcome.

## An approach to exploring the generation of experiences

This article explores the process of interpretation, the way in which an information processor can receive information and then determine what its identity is to that processor. This processing of information from an input to its identity can be quite complex. Most interpretation processes lead to simple identity information being acquired—what the input activity ‘is’ to the interpreter. But some processes of interpretation lead to information that includes an extra element within the meaning acquired. Consider two information processors. From the input activity the first receives, it is able to identify input patterns from its prior learning. It is able to receive an input pattern and recognize and identify it as the ‘thing’ it had learnt. This identity is semantic information, the meaning of the input pattern to the processor. The semantic information gained is what the input pattern ‘is’ to the processor.

**Figure 1:**
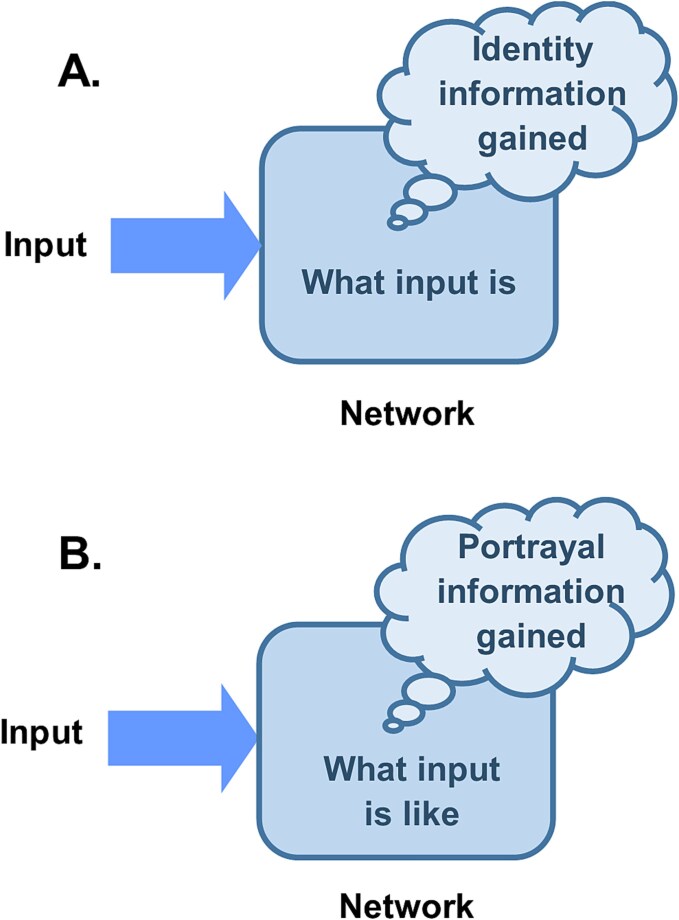
Acquisition of semantic information. The ‘cloud’ callout in this and subsequent figures is used to signify semantic information. (A) Semantic information gained by a network when it identifies an input as what it ‘is’ to the network. (B) Semantic information gained by a network when it identifies an input as what it is ‘like’ to the network.

Now consider a second information processor. This one is able to interpret its input not just as what it ‘is’ to the processor but what it ‘is like’ to the processor. This is of course purely hypothetical, and there is no need to think about how it might do that at this stage, but just consider a processor that has this capability. It is able to interpret its input not just as the thing it learnt from its prior experience but what that thing is ‘like’ to it. In this case, the information acquired by the processor is not just a straightforward identity of the thing, it must also include information about the way in which the thing presents itself to the processor. This extra information concerning the nature of the thing, ‘how’ the thing is to the processor rather than just ‘what’ it is, is defined here as portrayal information (Fig. 1). The receiver of this portrayal information would gain some sense of the subject of the portrayal. That sense of ‘how’ the thing seemed to the interpreter would indeed be the same as what we define as a quale. Using the example of interpreting the colour blue, identifying the input as ‘what it is’ just acquires the inner meaning ‘blue’. But if the brain were able to interpret this input as ‘what it is like’ then it would be acquiring the inner meaning ‘blueness’, what blue was like to it.

This discussion about the interpretation of ‘what something is like’ is just one example of the extra information that can be included in some interpretations. There are many others. The same extra subject information is acquired when interpreting something as how it appears to the interpreter, how it seems, how it is portrayed, how it is depicted. These identities all have the included information of appearance, seeming, portrayal, depiction. An interpreter going through such a process of interpretation acquires information that is more than just the identity of the thing, it acquires information about the nature of the thing.

So, the problem of linking neural activity to a phenomenal outcome is interpreted in this article as one of looking for a mechanism that is able to interpret input activity as not just what it ‘is’ to the interpreter but is able to acquire something about its nature as well, ‘how’ it seems to the interpreter. This is still quite a daunting exercise of course, but it does provide something to aim at in looking for neural mechanisms underpinning qualia. The rest of the article explores the information processing in a typical neural network with a view to showing how, in certain circumstances, this activity can include the process discussed above, and how it could lead to the emergence of qualia.

## Acquisition of information

### Information in the brain

In tackling the analysis of consciousness, approaches that consider information in the brain have been popular. The most influential of these is probably the integrated information theory, with its proposal that consciousness is the same as the cause–effect structure of a physical system that specifies a maxima of irreducible integrated information (Tononi 2008, Oizumi et al. 2014, Tononi et al. 2016). However, when it comes to theories that discuss information processing some authors are concerned that there is a confusion that sometimes exists between information and the physical realization of information (e.g. Pockett 2014). In neural tissues the two are closely linked. Orpwood (2017) tried to distinguish between the two by labelling them ‘semantic information’ and ‘physical information’, and explored the links between the two. In this characterization, physical information consists of all the trains of action potentials, the depolarizations caused by transmitter reception, the propagation of depolarizations throughout neurons, and all the physical phenomena that are the means for communication among the myriad of neurons, networks, and their interconnections. However, these physical activities are not in themselves the semantic information that underpins our mental world, the meanings that we acquire. As Pockett (2014) pointed out, they are just representations of that semantic information, and the means whereby it is communicated.

The pattern of action potentials in axons connecting to, say, the V4 area of the visual cortex, the colour processing area, is not in itself colour information. It is just the brain’s inner representation of that information. The information of colours arises from the way the neurons in V4 are able to interpret the incoming representations. Therefore, this article also uses the labelling of ‘physical information’ and ‘semantic information’ as it is felt that it is cognitively helpful to the argument being presented.

So, all the concepts and ideas that make up our inner mental world are elements of semantic information. They are the meanings represented by all the physical activity that we can observe in the brain, such as action potentials. The qualia that we experience, the component elements of the complex language of consciousness, are also a form of semantic information, as Chalmers (1996) pointed out.

### Acquisition of semantic information

**Figure 2:**
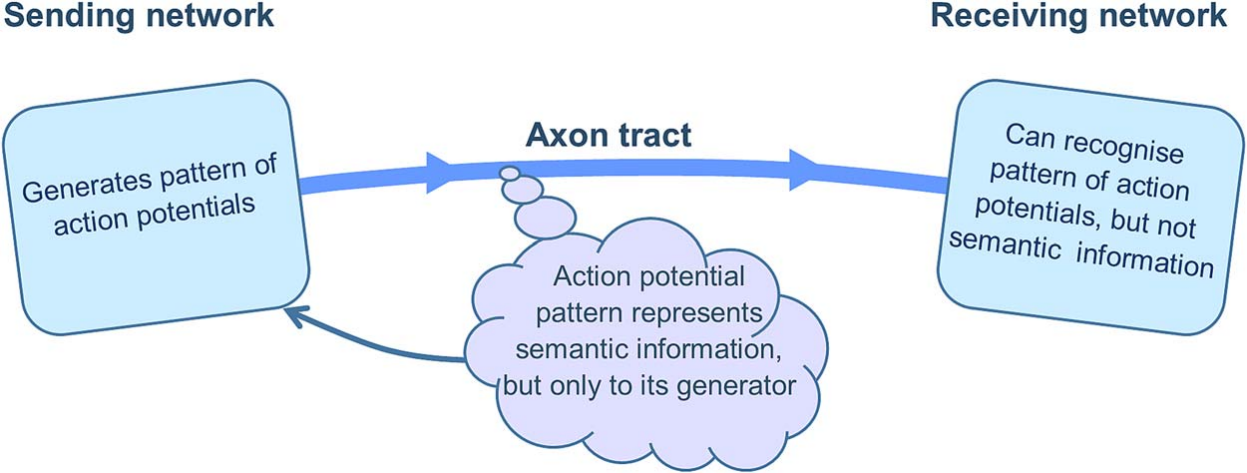
Receiving networks cannot recognize semantic information. To a network generating a pattern of action potentials, the pattern represents some semantic information. A network receiving them can only recognize the physical pattern and not the semantic information represented.

If all the observable physical activity in the brain is just a representation of its semantic content, how can that semantic information be realized? First of all, consider a network that is generating a pattern of action potentials and sending it onto other networks. This pattern of action potentials, physical information, represents some semantic information to its generating network. The receiver of that pattern of action potentials may be able to recognize the incoming pattern. But it can only recognize the physical pattern of action potentials. It cannot recognize the semantic information. Therefore, patterns of action potentials enable communication between a sender and a receiver, but they only directly represent semantic information to their sender (Fig. 2).

**Figure 3:**
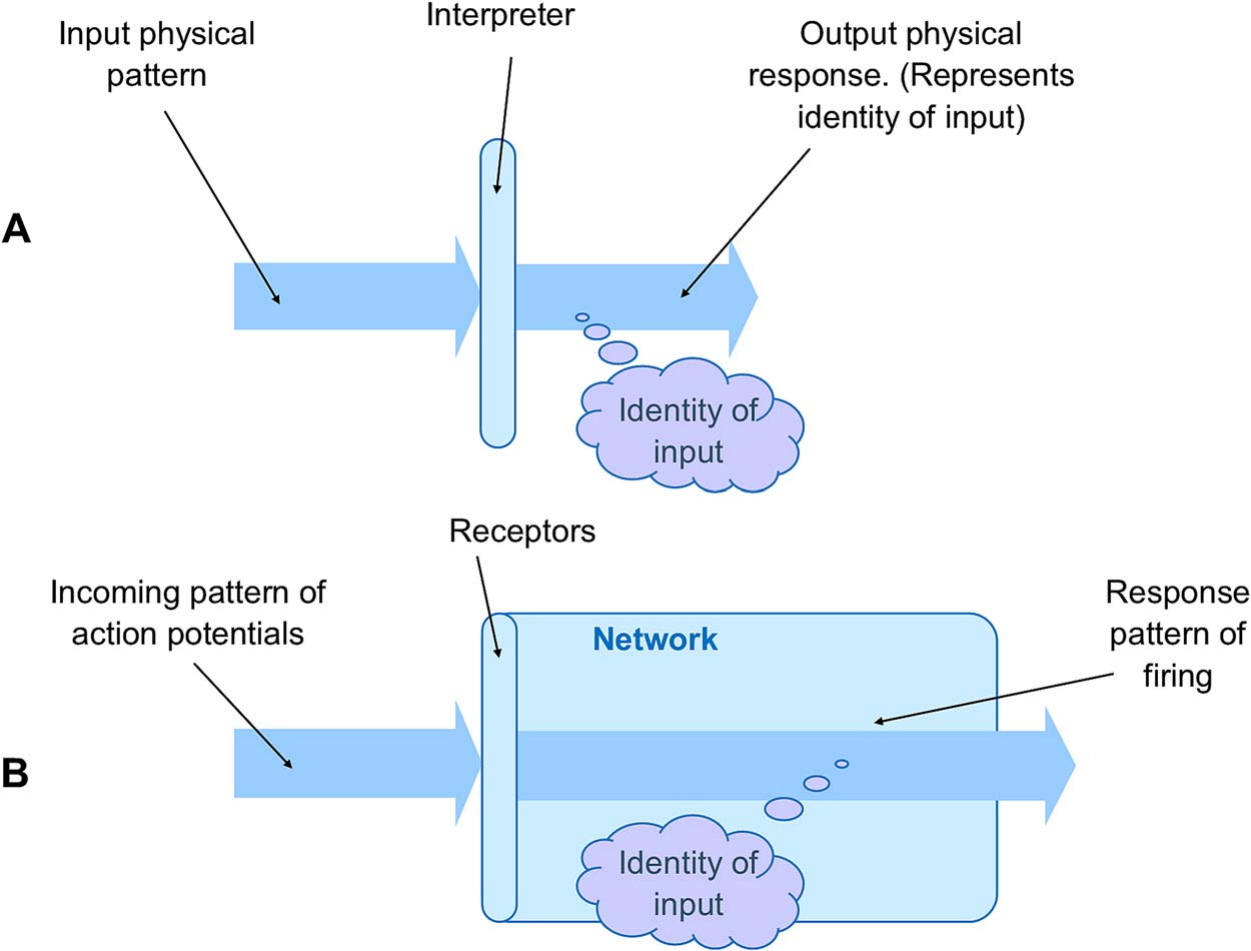
The process of interpretation. (A) If an input physical pattern can be recognized and identified, then its interpreter gains the semantic information of the input identity. (B) A network’s receptors can collectively act as an interpreter, acquiring the identity of an input pattern of action potentials, and that identity is represented by the network’s output pattern of firing.

So how then can semantic information be obtained by a receiver of physical information? Orpwood (2017) argued that semantic information can be acquired from physical information through a process of interpretation. It showed that the mechanism of interpretation involves three key physical elements: the input physical pattern received, the interpreter itself, and the physical response that is generated. If the input pattern interacts with the interpreter, and that interaction results in the interpreter generating some form of physical response, then the input has been recognized by the interpreter. If there is no response, then there is no recognition. If the form of the physical response is variable, and the same form is generated whenever a particular input is received, then the input has not only just been recognized by the interpreter, it has been identified as well. That particular input pattern has a specific identity to the interpreter. In addition, the form of the response represents the identity of the input to that interpreter. That identity is semantic information, and it is only available to the interpreter. The interpreter has acquired the semantic information of the identity of the input activity to it, and the physical response it generates represents that identity (Fig. 3A). The semantic information that the interpreter acquires depends on how the receptors in the receiving neural structures were configured, primarily during learning. The receiver has to use this prior learning, how it has been configured, to interpret the patterns it receives.

Consider the situation where the brain is attending to a patch of colour in its environment. As mentioned above, in this situation the visual area V4 receives physical information in the form of a pattern of neural firings. If the networks within V4 are able to respond in a typical way to that physical activity, then they will have acquired the semantic information of its identity to them. To these networks the physical activity they have received has a particular identity, and their response represents the identity to those networks. If physical information, such as a pattern of action potentials, is recognized and identified, then the outcome is the acquisition of semantic information. This is not to say of course that the semantic information is consciously experienced simply because this process has occurred. But the recognition and identification of the incoming physical activity leads to acquisition of semantic information, an identity (Fig. 3B).

It is felt that at the core of the ‘hard’ problem of consciousness is an understanding of the way that the brain’s physical activity can be recognized and identified to acquire semantic information. It is the problem of how the physical activity of neural firings, physical information, is identified in such a way as to enable the semantic information of qualia to emerge.

### Pattern information processing in single neurons

If recognition and identification are important for the acquisition of semantic information, then it is crucial to consider how these processes can be mapped onto the behaviour of neurons and networks of neurons.

It was predicted some years ago using modelling work that individual neurons should have the ability to recognize spatially distributed patterns among their inputs (Mel 1992, Orpwood 1994). Neurons are able to learn patterns of inputs spread over their dendrites. There is an increase in the strength of receptors activated by patterns of inputs during learning, and if a pattern is subsequently received that is similar to those on which it was trained, then this can lead to a reactivation of those stronger receptors and an increase in the depolarization of the neuron. If the input pattern is significantly similar to the training ones, then the depolarization initiated by these receptors can reach threshold and the cell will fire, thereby signalling recognition. But any pattern learnt will generate the same response, cell firing. Therefore, the neuron can recognize an input pattern and signal that recognition via its response, but it cannot differentiate between input patterns and thereby identify the input. The neuron on its own cannot acquire information about identity.

Pattern recognition is an important property of neurons, and it is useful to explore it a little further. At what point in the chain of events following the reception of a pattern of inputs does recognition take place? If the receptors were blocked, then no local depolarization would take place and no recognition would be possible. But if the cell-firing mechanism were blocked then there would still be a depolarization response, and that response would still surely indicate a recognition. However, the cell could not communicate that a recognition has taken place. So, as long as some form of physical activity occurred in response to the input pattern the cell would be capable of carrying out recognition. But for that recognition to be communicated elsewhere then the cell would have to fire an output.

### Pattern information processing in networks of neurons

What about the response from networks of neurons? Each of the neurons within the network would of course be able to respond in the way just described. The input pattern would be spread over many of the neurons in the network and this would lead to a complex network response. Like the individual pyramidal cells within the network, the network response is one of recognition. If there is a physical response following reception of the input pattern, then a recognition has taken place. The network can go further, however. If it receives an input pattern that is similar to one it has learnt, it will respond by generating a particular output pattern of firing. The network will always generate a typical output when it receives an input pattern similar to one that it has learnt. Therefore, such a network is not only able to recognize the input pattern, it is able to identify it as well. The response that the network generates when it receives a particular input pattern represents the identity of the input pattern to the network. This is the semantic information acquired, the identity of the input pattern to the network. Obviously this is not a conscious identification, but an identification has been made nevertheless. Therefore, individual neurons can provide a recognition, but a network can provide both a recognition and an identification. Thus, if identification is essential to the generation of qualia then the discussion should focus on the information processing of networks of neurons.

The focus on network behaviour reflects the conclusions of many others that the basic information processing entities in the brain are networks of neurons rather than individual cells. It is felt that it is ensembles of neurons that are key to the link between neuronal dynamics and their function in information processing (Harris 2005, Buzsáki 2010, Bharmauria et al. 2016). Harris (2005) argued further that the spatial pattern of firing activity in these neuronal ensembles is the fundamental currency of information processing in the cortex. In this article also the focus is on spatially distributed patterns of activity rather than temporal distributions.

## The interpretation process

### The network response and representations

Consider the response of networks to a learnt input in a little more detail. When a learnt input is received, it is interpreted and semantic information is acquired. That information is represented by the physical responses that are set in motion. Clearly, there are a number of different responses generated in the network, such as patterns of local depolarizations, patterns of action potentials, and patterns of transmitter release at the axon terminals*.* Each of these responses will lead to the next. This causal chain means that each of these individual events is part of the overall complex response of the network. They are all part of the network’s response to the interpretation of the original input pattern.

**Figure 4:**
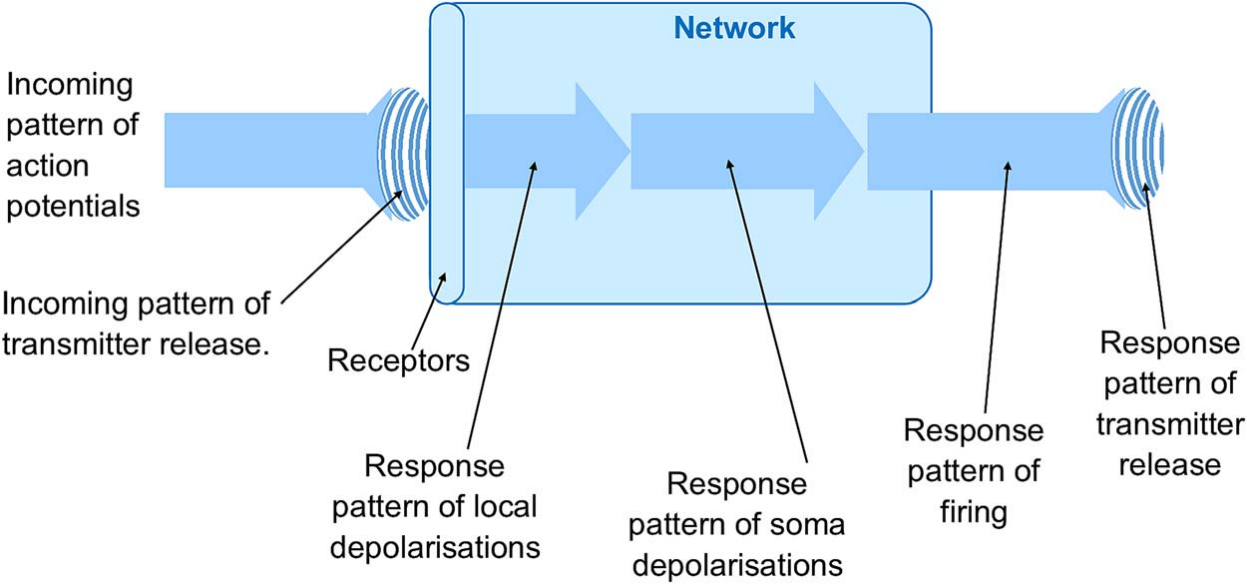
Network responses following an interpretation. Highly simplified diagram of the responses of the network’s neurons to an incoming pattern of action potentials, where the responses highlighted relate to the whole network. Following interpretation by the receptors, each pattern of responses leads to the next in a causal chain, and each pattern of responses represents the identity of the input to the network. To the network, the output pattern of transmitter release represents the identity of the input pattern of transmitter release.

The initial events in this causal chain of responses involve the network’s receptors. The structure of these receptors has resulted from the learning that the network has undertaken. The initial response of the network, the pattern of local depolarizations resulting from ions entering through receptor pores, only occurs following a particular input pattern, and therefore this initial response represents the identity of the input pattern. However, the network’s responses are all part of a causal chain. Events further down the chain from the receptor actions are also responses to events occurring at the receptors. Therefore, all these subsequent responses are also representations of the identity of the input pattern. This includes all the network’s responses up the final one of the pattern of neurotransmitters released at the axon terminals. Each of these responses are the ways in which the network represents the identity of the input that led to this chain of responses (Fig. 4).

The start of this process of interpretation is of course the input received. This input pattern is also a pattern of neurotransmitters received by the network from the incoming pattern of action potentials. So, the outgoing pattern of neurotransmitters released at the axon terminals is the network’s representation of the identity of the incoming pattern of neurotransmitters received by the network’s receptors.

The two processes taking place following the reception of an input pattern are interpretation and onward transmission of responses. There is a clear difference between the two. During interpretation, the receptor regions are receiving large numbers of different input patterns but only those activating receptor regions enhanced by the learning process will respond strongly. There is a selection process involved and a specific depolarization response is generated following a recognized input pattern. This is in marked contrast to the simpler process of the onward transmission of responses. For example, all the local depolarizations in the dendrites of the neurons in a network will lead to summed depolarizations of the network’s somas. There is no selection involved. It is just one set of events leading onto another set. And each of these subsequent events is a part of the network’s response following the interpretation of the input pattern, and all the events are part of the network’s representation of the identity of that input pattern.

### What is the identity acquired?

The physical response pattern and the semantic information it embodies are intimately linked. There are not two stages to the interpretation process where the network acquires semantic information and then generates a response to represent it. The semantic information is somehow contained in the response. There is of course a relationship between the physical information of the responses and the semantic information they represent. To the network, there are specific ways in which the physical structure of the representations relates to the identity. The representations are not just abstract symbols to the network, they have specific links to the identity they represent. So, to the network, the response patterns are more of a depiction of the identity information that they carry. It would seem that to the network the response structures that it generates are the various ways in which it depicts the input identity.

The interpretation process leads to the acquisition of an identity, but what is the identity acquired? All that the network is able to recognize is the pattern of activity that it receives. Its identity to the network is the identity of that pattern. The identity is ‘that pattern’ rather than any other. If the V4 region of the visual cortex gets an input pattern following exposure of the retinas to the frequency of light we call blue, then it can only recognize that pattern and interpret it as being ‘that pattern’. It does not at that stage interpret it with the label ‘blue’.

It is not the purpose of this article to explore the origin of the identities and the labels they acquire as a result of the brain’s interpretations, but it might be useful just to explore the origin of the blue identity a little further. In the developing brain, the label ‘blue’ must initially arise from the sound pattern of the word ‘blue’. The developing brain is shown an association between that sound pattern and the environmental exposure of a particular frequency of light. Therefore, there is pattern association learning taking place at some point higher in the cortex. When the brain is subsequently exposed to the visual input of the blue frequency of light, the output from the colour network could feed onto the higher network that learnt the pattern association, which could lead to an identity of the blue sound shape being recalled and an identity of the label ‘blue’.

## The impact of feedback

### Local feedback within cortical networks

The typical organization of local cortical networks includes a very large amount of local feedback. Around 95% of the inputs received by pyramidal cells come from other cortical cells, with around 50% coming from other local pyramidal cells (Abeles 1991, Stepanyants et al. 2009). When an input pattern is received by the network and recognized, there is a typical output pattern of firing generated, as discussed above. A large proportion of this output firing is fed directly back to the local network via axon collaterals, and provides a new input pattern for the cells receiving it (Braitenberg and Schultz 1991). The feedback input pattern is interpreted by the network, in the same way as the original pattern coming from outside of the network. This feedback input is a new pattern for the network to interpret, in accordance with how the new receptors have been configured.

### Interpretation of feedback

Assume for now that the new receptors receiving the feedback are able to respond to this new input. Following direct feedback, the new input pattern received is the pattern of neurotransmitters just released at the axon terminals in response to the original input. But to the network that pattern of neurotransmitters is a representation of the identity of the original input. If the network is able to interpret that feedback input, then it will be interpreting its own representation. So, following direct feedback the network will be interpreting an input pattern that, to that network, is its depiction of the original input.

**Figure 5:**
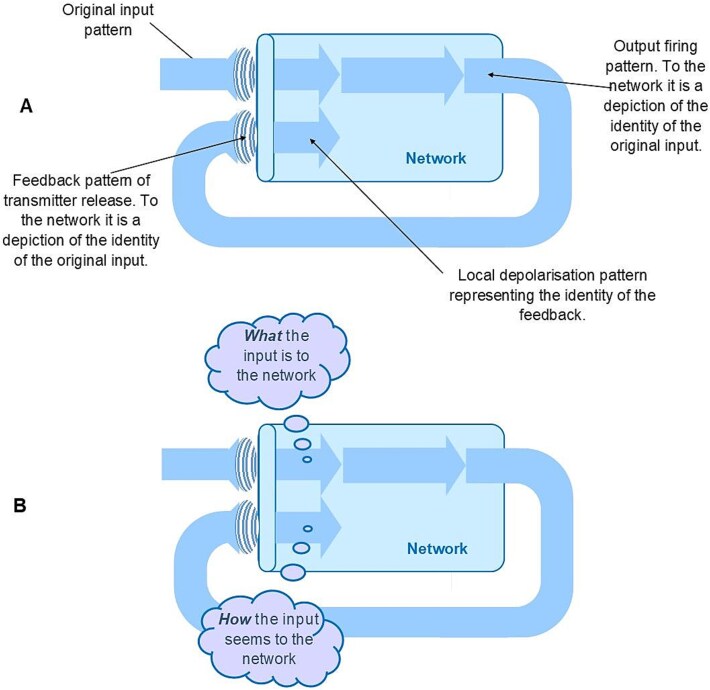
The network’s interpretation of direct feedback. (A) The identity of the original input pattern is represented by the pattern of the transmitter released on feedback. The network can only interpret the structure of this input. This structure is the way the network depicts the previous identity, and the new identity acquired is represented by the new local depolarization pattern. (B) The semantic information acquired. The original identity acquired is ‘what’ the input pattern is to the network. Following direct feedback, the new identity acquired is that of the way that the network depicted the original identity, ‘how’ the input pattern seems to the network.

If the network is able to interpret the feedback input, then it will generate a new set of responses, and those responses will represent the identity of the feedback to the network. But what is that identity? The receptors can only identify the physical information they receive, the structure of the feedback. To the network, however, the structure of the feedback is the way it depicts the identity of the original input. Therefore, the new identity acquired from the interpretation of the feedback, what the feedback ‘is’ to the network, is the way it depicts the previous identity. This is more than just a straightforward identity: it includes descriptive information. It is how the network has depicted the previous identity. For a straightforward identity, the semantic information gained by the network is a kind of label, what the input ‘is’ to it. For the complex identity acquired on feedback, the semantic information gained by the network is depictive, how the input ‘seems’ to it (Fig. 5).

Consider again the example of identifying activity resulting from the transduction of a particular frequency of light by the retina. To a network in the V4 part of the visual cortex, this pattern could be learnt to have a particular identity to it. It is just a simple identity and no further information is acquired. At this stage, there is no label we can apply to the initial identity other than it is ‘that pattern’. However, as argued, that abstract pattern could be learnt to have a label such as ‘blue’ higher in the cortex. The colour identity acquired by V4 is represented by the response that the network generates. If that response is fed directly back to the network, then it will be interpreting an input pattern that is the network’s depiction of blue. The identity gained, what the structure ‘is’ to the network, is the way in which the network depicts blue. It is ‘how’ blue is to the network, what we would call the ‘quality of blueness’.

The complex identity discussed above is similar to the more complex identities discussed earlier such as identities of ‘how it seems’, ‘how it appears’, or ‘what it is like’. These are identities of not just ‘what’ a thing is, but identities of its nature. For a network acquiring such an identity, it is gaining semantic information concerning ‘how’ something is to it rather than simply ‘what’ it is. Such an identity has descriptive content, which must manifest in some way. It is suggested that the semantic information gained by the network when it interprets the identity of the way it depicts something is what we would interpret as a quale. However, it is accepted that even though the network has information concerning how the original input seems to it, it could still be questioned how this leads to an experience. But it is felt that when interpreting the feedback something is portrayed to the network in a descriptive manner. The network is gaining information ‘about’ something rather than just stating its existence. This about-ness information seems very close to the experience of a quale.

The important role of metarepresentations in HOTs is partially reflected in the theory proposed here. In HOTs, a higher-order representation of a mental state is thought to enable consciousness (e.g. Lau and Rosenthal 2011). However, the metarepresentations generated in the theory presented here are the result of networks interpreting their own direct feedback. Therefore, there is a difference in that the metarepresentations generated by the networks are the result of them interpreting their own activity. They are generating representations of their own representations. It is these metarepresentations themselves that are argued to represent the complex identities interpreted from feedback. It is they that represent qualia.

The semantic information acquired by the network will persist as long as the response persists. This duration of course would only be a few milliseconds for a single input to the network. If there were repeated inputs, then the duration would be much longer and would be maintained while the input was repeated. If an attractor developed, then the identity acquired would remain the same while the attractor state remained, and would lead to a much longer semantic response.

## Conditions needed for qualia generation

### Generation of feedback

The conclusion from the argument above is that a key condition for qualia generation is the generation of direct feedback in pyramidal cells. For feedback to occur, the neurons involved must be able to fire action potentials following the local depolarizations that result from their initial receptor activity. This firing is inevitably going to be influenced by the balance of excitatory and inhibitory inputs received by the neurons involved. Pyramidal cells in the sensory cortex receive such tonic inputs from feedforward and feedback connections, as well as local recurrent connections (Shepherd 2004). For neurons with little background depolarization, or with hyperpolarization from inhibitory inputs, the local depolarizations generated by the primary receptors may go no further than summing together in the soma, but at a level below firing threshold. Therefore, tonic depolarization could be a crucial factor in determining which networks are able to respond by firing, and therefore possibly go on to generate qualia.

The structure of pyramidal cells is particularly well organized for receiving top-down modulatory inputs, mostly to their apical dendrites (Cauller 1995). Attentional inputs from the prefrontal cortex could act in this manner and provide the cell with a background depolarization that makes them much more responsive to the inputs received by dendrites nearer the soma (Moran and Desimone 1985, Phillips et al. 2016). Additionally, there is some speculation that recurrent feedback from networks higher in the sensory hierarchy could also provide a modulatory influence (e.g. Lamme 2010, Wyatte et al. 2014).

### Recognizing the feedback

The network response to direct feedback would only occur if the receptor regions of the network were able to recognize it. The feedback receptors would have to have been through a learning process to be responsive in the way described. One of the ways that feedback receptors may be able to do this is through the process of autoassociative learning. In this process, the primary response of the network is used to train the feedback receptors (Hopfield 1982, Rolls 2016). It has been suggested that autoassociative learning confers many useful properties to networks, such as generalization and pattern completion (i.e. networks are able to generate a normal response to an input pattern even if that input is only part of the original pattern; e.g. Kohonen 1984).

If the network has undergone autoassociative learning, then a strong response to the feedback would result. The interpretation of the feedback would lead to the acquisition of semantic information depicting the original identity. With attentional facilitation, this response could lead to further firing of the network’s neurons to generate a new output. This new output would be very similar to the previous one and would be fed back again. Therefore, the network could settle into cycles of the same activity, forming an attractor, where the output from the network remains unchanged for the duration of the feedback cycles. Once an attractor has been established, it might seem that the network’s interpretations could become recursive, i.e. the identity of the feedback becomes the identity of a representation of that identity. But that is not felt to be the case. Once an attractor has formed, the firing response of the network on each cycle of activity would be very similar to the original network response. To the network that response has been established as being a representation of the original input identity, and it would continue to represent that identity throughout the attractor cycles. The qualia that are acquired are the result of the initial fizz of activity within the network following reception of the direct feedback.

## Role of attention in the behaviour proposed

It was suggested above that attention could have a strong modulatory impact on the responses of cortical networks. There has long been a debate about the link between attention and consciousness. Some have argued that consciousness is not dependent on attentional focus (Koch and Tsuchiya 2007, van Boxtel et al. 2010) but there is much evidence to indicate the opposite. A review of the topic (Bor and Seth 2012) concluded that all conscious events require attention in some form, and that attention plays a critical role in selecting conscious content. There is good evidence for this role. Both lesion (e.g. Del Cul et al. 2009) and transcranial magnetic stimulation (Turatto et al. 2004, Rounis et al. 2010) studies have shown conscious detection of sensory stimuli to be markedly affected by interruptions of prefrontal activity. In an electrocorticogram (ECoG) study exploring masking, prefrontal activity was strongly linked to conscious detection of words (Gaillard et al. 2009). Prefrontal activity reduction following administration of an anaesthetic has been shown to reflect loss of conscious detection (Davis et al. 2007). Inattentional blindness has long been thought to indicate the need for attention in consciousness (Mack and Rock 1998). Attention has also been shown to be necessary for conscious perception of visual summaries of colour and size (Jackson-Neilsen et al. 2017). And change blindness (Rensink et al. 1997) is also a good example of the importance of attention in conscious awareness. It seems that attention acts as the selecting mechanisms for conscious content (e.g. Dehaene and Changeux 2011).

Therefore, there is much evidence that attention is an important ingredient in the attainment of consciousness. It was argued above that tonic depolarization of networks can enable direct feedback to occur, and may also encourage the development of attractor behaviour. This background depolarization could well arise from attentional activation. So, this article suggests that the role of attentional focus is to encourage the development of local direct feedback in lower networks following a competitive selection process involving frontal regions (see model of attention in Knudsen 2007). This feedback would enable the networks activated by this attentional focus to experience qualia. The attentional focus would provide a means for selecting which networks in the lower cortex were brought into consciousness.

## Discussion

The theory proposed in this article revolves around the development of direct local feedback, particularly in the cortex. But the argument presented would apply to such activity anywhere in the brain, including subcortical structures. For example, the principal cells in the basolateral complex of the amygdala are heavily interconnected. Like the cortex, these principal cells comprise about 80% of the cells present. These cells have multiple axon collaterals that provide numerous excitatory connections to other principal cells, and to inhibitory interneurons (Duvarci and Pare 2014). Such microcircuitry would appear to be well suited to the development of local feedback in this nucleus, and could well underlie emotional qualia.

In contrast, the cerebellum has long been claimed to have no role in conscious activity. It is interesting that although the actions of the input fibres to the Purkinje cells are excitatory, and their outputs engage in recurrent feedback, this feedback is only inhibitory. In contrast to the cerebral cortex, there are no pathways that enable excitatory recurrent activity (Shepherd 2004). As was pointed out long ago (Eccles et al. 1967), this means sustained activity within cerebellar circuits in response to an input is not possible. According to the theory presented here, this lack of local direct feedback activity within local cerebellar circuits may explain why there is no conscious awareness of cerebellar activity.

Considering consciousness at the whole-brain level underlines one of the limitations of the theory presented. This article purely deals with the issue of how the physical information of a pattern of action potentials received by a network can be transformed into the inner portrayal of a quale. Terms such as ‘recognition’ and ‘identification’ that are discussed at length in this article purely relate to mechanisms taking place at the individual network level. The qualia that are described are purely those linked to the activity of individual networks. Clearly, the brain-wide conscious state depends on how individual qualia are choreographed so as to arrive at an overall concluding conscious state.

The other limitation of the theory discussed so far is that it has only really considered single presentations of an input pattern. In practice, of course, there is likely to be a train of inputs arriving at networks following exposure to a sensory stimulus. Given the complexity of tonic inputs to the pyramidal cells involved, it may well be that repeated exposure to the input pattern is required to overcome background inhibition and generate an initial response. Certainly, it is widely reported that it takes some time for attentional feedback to influence primary sensory activity (e.g. Martinez et al. 2001, Noesselt et al. 2002), and that even recurrent feedback from networks higher in the sensory cortex can take over 100 ms (e.g. Wyatte et al. 2014). If this tonic input is necessary to enable pyramidal cells to overcome the inhibitory background, then it is likely that a sensory input would have to continue for some time before it can lead to a response from sensory cortical networks. [Although, in visual masking experiments, Rolls has reported that only 30 ms is required for an identification (Rolls and Tovee 1994)]. However, these considerations do not change the basic hypothesis that qualia arise when sensory networks respond to an input pattern, and when that response is then directly fed back to the network again.

Once available as a quale, the actual feel of complex identities is very difficult to relate to the location of the network involved, or to the particular structure of the response interpreted as a quale. However, different input patterns to the same network may well lead to qualia with a similar ‘feel’. Consider different input patterns to V4 that have arisen from the exposure of the eye to different wavelengths of light. These patterns may well lead to distinctive qualia being experienced, but the common network involved may lead these experiences to have a similar ‘feel’, what we would describe as colours. Conversely inputs to completely different networks, say to the amygdala, will have their own completely different ‘feel’ once qualia are generated. These emotional qualia would be ‘felt’ to be different to the colour qualia sensed by V4 (Orpwood 2010).

One of the outcomes of autoassociative learning being involved in qualia generation is that the networks engaged in such activity are likely to develop attractor behaviours. There has been an implicit assumption for some 20–30 years that many cognitive functions involve attractor behaviour (e.g. Hopfield 1982, Amit 1989, Rolls and Treves 1998), and a possible link between attractors and consciousness has been suggested by a number of authors (e.g. Mathis and Mozer 1996, Tsodyks 1999, Mozer 2009). Humphrey has long argued that consciousness results from a large-scale attractor between sensory and motor areas (Humphrey 1992, 2016). Capaday et al. (2013) concluded that the large amount of recurrent collaterals in the motor cortex would enable the generation of attractors to represent kinetic data. Herzog and his colleagues have long presented an argument that environmental inputs to the cortex are initially processed unconsciously, only to reach consciousness when the processing has reached some kind of consensus, with this consensus reflected in the settling of an attractor state (e.g. Herzog et al. 2016). The likely development of attractors as a result of the interpretation of direct feedback, and the acquisition of qualia, would support these hypotheses of a link between attractors and consciousness.

As with any theory relating to mechanisms of qualia generation, any proof would require the involvement of human report. The proposed involvement of individual local networks in qualia generation makes the predicted network activity difficult to explore using noninvasive monitoring techniques such as EEG, MEG, and fMRI*.* It is likely that electrocorticogram (ECoG) investigations would be necessary to check that activity in local networks is correlated with qualia generation, and to detect attractor activity in those networks.

However, it is clear that sensory inputs can be processed unconsciously. The theory presented would suggest that in these situations the tonic inhibition received by sensory networks is preventing direct feedback responses but still allowing the primary response. This differential sensitivity to inhibition could be used to explore the mechanisms involved if it were possible in some way to control the inhibitory inputs. If the cortical colour networks received increased inhibition while the subject focussed on, say, a coloured shape on a screen, there would come a point where the primary input would still elicit a response from the colour network but the feedback would not. The subject would not be able to report a colour experience but could still report the shape. However because the primary response still occurred then the colour identification would have occurred and would be fed forward up the sensory hierarchy. If the subject was asked to guess the colour of the shape, their response should be significantly above chance. The primary response had occurred without qualia, but the subject still had implicit knowledge of the colour. Such dissociations have a precedent in studies of the unconscious perception that occurs in blindsight (Weiskrantz et al. 1974). These experiments would of course not be conclusive without data on the actual pathways involved, but they would provide some confidence that the mechanism was a possible one.

## Data Availability

There is no new data associated with this article.
